# Methods to label, image, and analyze the complex structural architectures of microvascular networks

**DOI:** 10.1111/micc.12520

**Published:** 2019-01-17

**Authors:** Bruce A. Corliss, Corbin Mathews, Richard Doty, Gustavo Rohde, Shayn M. Peirce

**Affiliations:** ^1^ Department of Biomedical Engineering University of Virginia Charlottesville Virginia

**Keywords:** blood vessels, image analysis, image quantification, microvasculature, vascular network, vessel architecture

## Abstract

Microvascular networks play key roles in oxygen transport and nutrient delivery to meet the varied and dynamic metabolic needs of different tissues throughout the body, and their spatial architectures of interconnected blood vessel segments are highly complex. Moreover, functional adaptations of the microcirculation enabled by structural adaptations in microvascular network architecture are required for development, wound healing, and often invoked in disease conditions, including the top eight causes of death in the Unites States. Effective characterization of microvascular network architectures is not only limited by the available techniques to visualize microvessels but also reliant on the available quantitative metrics that accurately delineate between spatial patterns in altered networks. In this review, we survey models used for studying the microvasculature, methods to label and image microvessels, and the metrics and software packages used to quantify microvascular networks. These programs have provided researchers with invaluable tools, yet we estimate that they have collectively attained low adoption rates, possibly due to limitations with basic validation, segmentation performance, and nonstandard sets of quantification metrics. To address these existing constraints, we discuss opportunities to improve effectiveness, rigor, and reproducibility of microvascular network quantification to better serve the current and future needs of microvascular research.

**Abbreviations**
3D: three‐dimensional
ASMA: alpha smooth muscle actin
CD31: cluster of differentiation 31
CD34: cluster of differentiation 34
Col‐IV: collagen‐IV
CRISPR: clustered regularly‐interspaced short palindromic repeats
DAPI: 4′,6‐diamidino‐2‐phenylindole
DsRed: a red fluorescent protein
EGFP: enhanced green fluorescent protein
EMCN: endomucin
EP: endpoints
FLK‐1: fetal liver kinase‐1
FLT‐1: FMS‐like tyrosine kinase‐1
FPALM: photo‐activated localization microscopy
GFP: green fluorescent protein
HSC: hematopoietic stem cells
IB4: griffonia simplicifolia lectin I isolectin B4
MSC: mesenchymal stem cells
NG2: neural/glial antigen 2
OCT: optical coherence tomography
PDGFRβ: platelet‐derived growth factor receptor beta
PECAM: platelet endothelial cell adhesion molecule (CD31)
RAVE: rapid analysis of vessel elements
RFP: red fluorescent protein (eg, DsRed)
SSR: sum of squared residuals
Tie1: tyrosine kinase with immunoglobulin‐like and EGF‐like homology
Tie2: angiopoietin‐1 receptor
VAF: vessel area fraction
VE‐cadherin/VE‐cad: vascular endothelial cadherin
VEGFR: vascular endothelial growth factor receptor
vWf: Von Willebrand factor

## 1 INTRODUCTION

**Figure 1:**
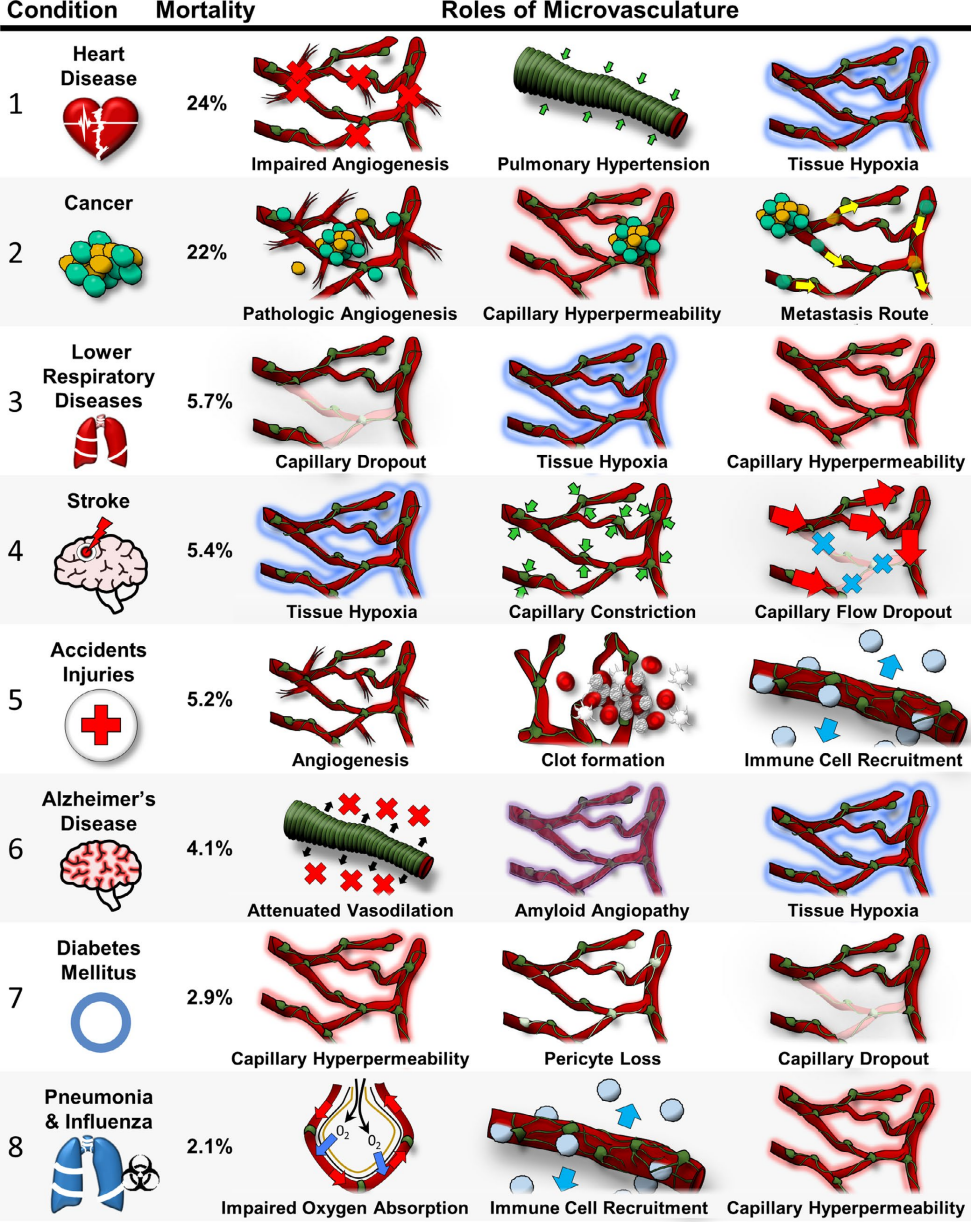
The significance of the microvasculature in top causes of death and disease in United States. Top eight classes of fatal disease or injury with the fraction of annual deaths in the United States. Included with each malady are three highlighted fundamental roles the microvasculature plays with initiation, maintenance, or treatment (see main text for references)

The microvasculature plays a plethora of key roles in maintaining tissue homeostasis, including modulating oxygen transport,^1^ nutrient delivery, inflammation response,^2^ and wound healing.^3^ Structural changes to the microvascular architecture have been shown to profoundly regulate these fundamental biologic processes.^4^ Therefore, characterization of the complex changes in spatial structure of the microvascular architecture gives a better understanding of the roles microvessels play in pathogenesis, maintenance, prevention, and amelioration of diseases. Indeed, the importance of the microvasculature has long been appreciated in diseases such as small vessel disease,^5^ coronary microvascular disease,^6^ and the abundance of complications associated with diabetes.^7^ However, recent research has indicated that the microvasculature also plays key roles in the top eight causes of death in the United States^8^ (Figure 1) and many others, including (1) heart disease: impaired infarct wound healing,^9^ reduced oxygenation,^10^ pulmonary hypertension in pre‐capillary and post‐capillary vessels^11^; (2) cancer: pathological angiogenesis,^12^ enriched microvessel permeability,^13^ significant route for metastasis^14^; (3) lower respiratory disease: capillary dropout,^15^ reduced muscle oxygenation,^16^ airway rigidity from vasodilation^17^; (4) stroke: impaired microvascular flow patterns^18^ and reduced oxygenation,^19^ pericyte constriction of capillaries,^20^ dropout of functioning capillaries^21^; (5) unintentional injuries: angiogenesis,^22^ clot formation,^23^ immune cell recruitment^24^; (6) Alzheimer's: attenuated vasodilation response,^25^ amyloid angiopathy,^26^ and tissue hypoxia^25^; (7) diabetes mellitus: capillary permeability, pericyte dropout, capillary dropout^27^; and (8) pneumonia and influenzas: capillary permability,^28^ immune cell recruitment,^28^ and impaired lung oxygen transport.^29^ Additionally, the microvasculature is recognized as one of the most promising routes of drug delivery^30^ by enabling direct targeting of microvascular endothelial cells with intravascularly injected drugs to exert profound therapeutic effects in disease conditions.^31^ The overall import of the microvasculature in biomedical research is quickly approaching that of the nearly ubiquitous roles that the immune system plays in basic organismal processes^32^, ^33^ and disease development,^34^ and future research focused on microvascular structure, function, and adaptations promises profound opportunities for curing human disease.

In this review, we highlight new key developments and survey contemporary and classical models of the microvasculature, along with techniques to label and image microvessels at high resolution where the complete microvascular structure is captured. Therefore, microvascular research that fails to resolve the smallest‐sized vascular structures is omitted or given less emphasis, such as fundus imaging of the retina^35^ and other clinical imaging methods. Although a subset of the modalities covered can yield 3D images, we focus on analysis of 2D projections of 3D vessel networks since it can be universally applied to all microvascular imaging modalities, 2D representations of 3D networks retain much of their information,^36^ and 2D methods for quantification of vessel architecture can be extended to three dimensions,^37^ although we do comment on the potential pitfalls of using 2D metrics to characterize 3D microvascular structures. Furthermore, many of the 3D modalities for microvascular imaging have reduced axial resolution compared to lateral, and practical considerations of acquisition time usually lead to further reduced axial sampling.^38^ The currently available programs to analyze and quantify microvascular structures are also covered, along with constructive proposals for improvement in this area. While each topic covered could be a detailed examination on its own, this review is meant to offer a basic orientation of the technological options available for microvascular research and a perspective on analytical techniques to increase scientific rigor as science faces an ongoing crisis in reproducibility.^39^

## 2 MARKERS AND MODELS TO STUDY MICROVASCULAR NETWORK ARCHITECTURE

The study of the complex architecture of microvasculature requires proper labeling and visualization of microvessels, using either a marker for particular cell types, unique basement membrane constituents, and/or labeling perfused microvessels via the intravascular injection of a dye or fluorescently tagged antibody to visualize blood flow through microvessel lumens.^40^, ^41^, ^42^ All of these labeling methods provide a means of contrasting microvascular architectures with the surrounding tissue when paired with a suitable imaging modality. The particular choice of vascular marker and imaging method should be carefully evaluated based on the biological questions being examined and determined based on requirements for resolution, signal to noise, tissue penetration depth, imaging location in terms of in vivo/ex vivo, and labeled cell specificity.^38^ The relative importance of the various labeling and imaging considerations for microvascular visualization depends on the nature of the research and biological questions asked. For example, with investigations focusing on angiogenesis and subsets of vessel types, cell labeling of specific subpopulations is essential, while for studies characterizing blood flow, accurate vessel diameter and connectivity between vessel segments have greater significance. Moreover, effective pairing of these technologies with a particular imaging approach requires an understanding of the fundamental strengths and weaknesses of the options available.

### 2.1 Markers of microvasculature

**Figure 2:**
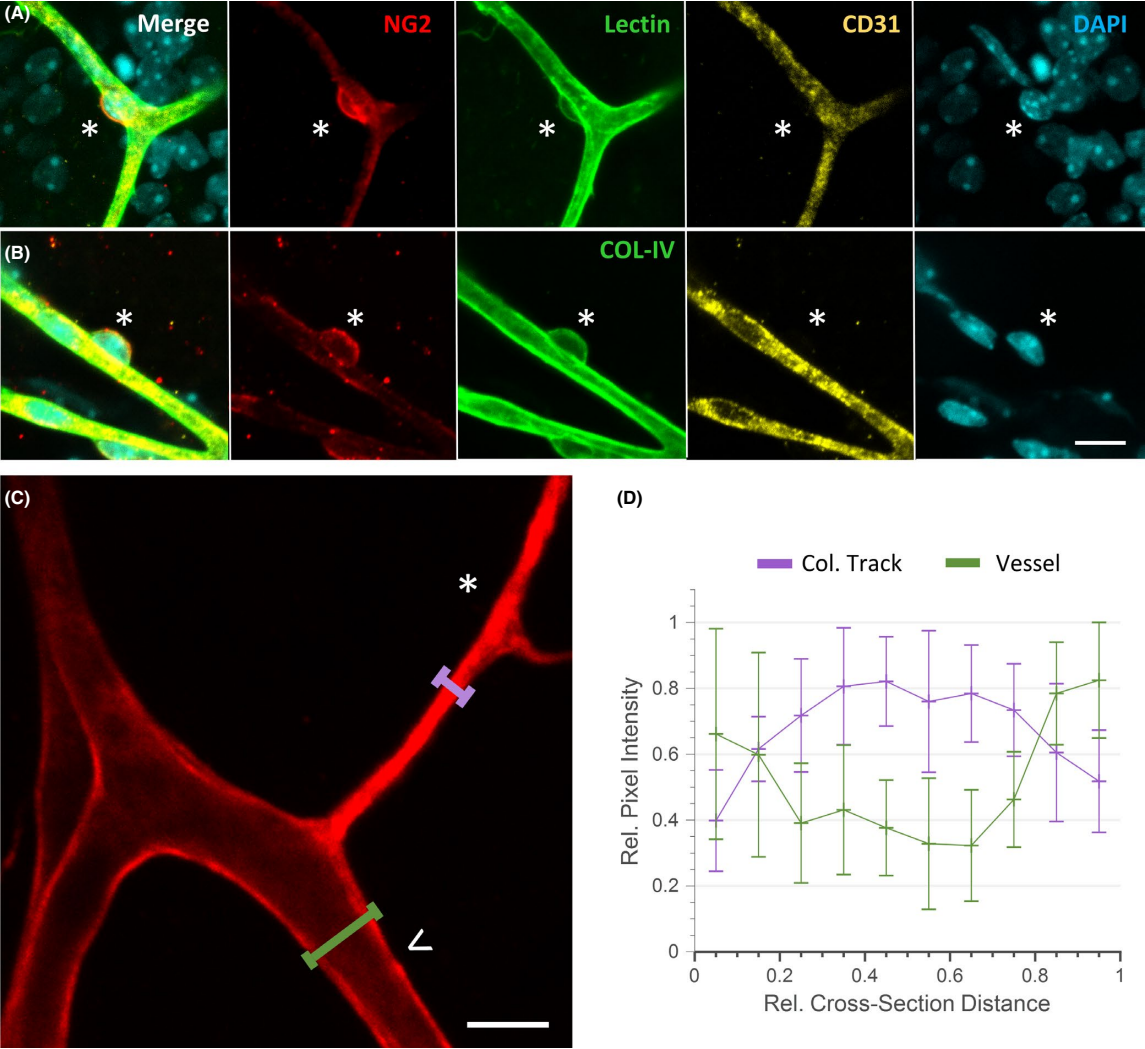
Both endothelial cells and pericytes share markers used in the literature for labeling endothelial cells, and Col‐IV tracks, assumed to be regressed vessels, lack a pixel intensity profile indicative of a lumen. (A) Retina capillary with pericyte (NG2, red), IB4 lectin (green), endothelial cells (CD31, yellow), and cell nuclei (DAPI, cyan). (B) Retina capillary with pericyte and endothelial cells labeled with Col‐IV (green; scale bar 10 μm). (C) High‐resolution image of Col‐IV off‐vessel track (star) and lumenized blood vessel (arrow; scale bar 5 μm). (D) Comparison of Col‐IV relative pixel intensity profile across cross‐section of blood vessels and collagen tracks (*P* = 8.11E‐6, 2‐way analysis of variance, N = 10 vessels and tracks, error bars are standard deviation)

A critically important aspect of studying blood vessels is carefully tailoring biological interpretations and conclusions to appropriately correspond to the specific cell types or structures visualized (Table 1). An example that illustrates incongruity between data and interpretation is when vessels are labeled via perfusion of fluorescent dye^43^ and general conclusions are made about vascular remodeling, disregarding changes in structure of nonperfused vessels, vessel neosprouts, and regressing vessels.^44^ Even with specifically worded conclusions, focusing on findings that only quantify portions of the microvascular architecture represents an incomplete analysis and may omit significant remodeling events. Another key example is the use of superfused IB4 lectin, a marker commonly used to label blood vessels that also labels pericyte^45^ and macrophages^46^ (Figure 2A). Especially in development, many papers prematurely conclude changes occur in vascular architecture based on lectin staining^47^, ^48^ while omitting the issue that a mix of cell types are labeled, especially with the inability to differentiate structures between pericyte and endothelial cells. Studies that use Col‐IV staining to quantify microvascular architecture^49^ have similar shortcomings, labeling not just blood vessels^50^ (Figure 2B), but other cell types such as pericytes and fibroblasts.^51^ Additionally, Col‐IV also marks thin bridges between capillaries previously referred to as Col‐IV sleeves,^52^ string vessels,^53^ and acellular capillaries^54^ in various tissues such as retina,^54^ brain,^55^ and muscle. Especially in the retina, this feature is interpreted as a sign of collapsed or regressed vessels, yet this has never actually been established. There is a clear separation between the two structures in thickness (Figure 2C) and with the cross‐sectional pixel intensity profile between lumenized vessels and Col‐IV tracks (Figure 2D), with a lack of structures found in an intermediate or transitioning phenotype. An alternative hypothesis would be pericytes extending off‐vessel processes^56^ and secreting Col‐IV.^57^ For instances where the cell type responsible for an immunostained structure is not established with confidence, we caution interpreting results are cell‐type specific, even if previously stated in the literature.

**Table 1:** Markers of the Microvasculature

***Diagram***		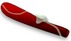	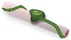	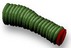	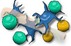
Name	**Type**	**endothelial cells**	**pericytes**	**smooth muscle cells**	**Other**
**PECAM/ CD31**	Surface^136^	✓^137^	✗^138^	✗^139^	Platelets, T‐cells. leukocytes^137^
**IB4 Lectin**	Var.^140^, ^141^	✓^46^, ^142^	✓^45^	✓^143^	Macrophages^144^, microglia^46^, monocytes^145^
**Col‐IV**	ECM^40^	✓^146^	✓^147^	✓^148^	Fibroblasts^149^, MSCs^150^
**Laminin**	ECM^151^	✓^152^	✓^152^	✓^152^	Fibroblasts^153^, epithelial cells^154^
**CD34**	Surface^136^	✓^155^	✗^156^	✗^157^	MSCs, HSCs, muscle satellite cells, fibrocytes^158^
**VE‐cadherin**	Surface^138^	✓^138^	✗^159^	✗^160^	None^161^
**EMCN**	Surface^162^	✓^163^	✗^164^	✗^164^	Putative hematopoietic progenitor cells^165^
**vWf**	Internal^166^	✓^167^	✗^167^	✗^168^	Megakaryocytes^166^
**aSMA**	Internal^169^	✗^169^	✓^170^	✓^171^	Fibroblasts^172^, perisinusoidal cells^169^
**MYH11**	Internal^173^	✗^173^	✓^174^	✓^175^	None^174^
**NG2**	Surface^176^	✗^176^	✓^157^	✓^156^	Microglia^170^, macrophages, oligodendrocyte porogenitors^177^
**N‐Cadherin**	Surface^178^	✓^179^	✓^159^	✓^180^	MSCs^181^, fibroblasts^159^, osteoblasts^159^
**Desmin**	Internal^182^	✗^168^	✓^183^	✓^156^	Interstitial cells, muscle satellite cells^158^
**PDGFRβ**	Surface^184^	✗^185^	✓^156^	✓^156^	MSCs^156^, fibroblasts^156^ **,** some neurons^156^
**Perf. PECAM**	Surface^186^, ^187^	Perf.^186^	✗^138^	✗^138^	Platelets^188^, leukocytes^158^
**Perf. Neuro‐Trace**	Internal^189^	✗^189^	Capillary^186^	✗^189^	Other neuronal cells when *fixed* (brain)^189^
**FITC**	Passive^42^	Perf.^42^	✗^42^	✗^42^	None^42^
**Tie2**	Surface^190^	✓^64^	✓^*^ 64	✓^*^ 64	Monocytes, macrophages^67^, HSCs^191^
**Dextran**	Passive^192^	Perf.^192^	✗^192^	✗^192^	None^193^
**VEGFR2, FLK‐1**	Surface^194^	✓^194^	✗^139^	✗^139^	HSCs^195^, macrophages^196^, neurons^197^
**VEGFR1, FLT‐1**	Surface^198^	✓^198^	✗^199^	✗^199^	HSCs, macrophages^196^, neurons^197^

EC, endothelial cell, ECM, extracellular matrix; HSC, hematopoietic stem cell, MSC, mesenchymal stem cell, Perf., perfused; PC, pericyte, SMC, smooth muscle cell; Var., various. **Labeling**: yes (✓), no (✗).

*Note:* Table shows general trends, there are exceptions with specific tissues, species, and disease conditions. ^*^See main text.

Another challenge for marking the microvasculature is identifying effective markers for PCs. Pericytes have no well‐established cross tissue exclusive marker, making them hard to target for analysis,^58^ although recent system‐level analyses have revealed novel candidate markers that await confirmation.^59^ A major point of contention with pericytes markers includes consensus with ASMA expression in pericytes, which is thought to be absent in pericytes on capillaries throughout some tissues, such as retina.^60^ However, recent evidence indicates that this may be a product of how tissue samples are processed: In the retina, it was shown that if standard fixation techniques are used with ASMA staining, capillary pericytes are ASMA‐negative,^61^ but if the samples were snap frozen with methanol fixation to prevent actin depolymerization, at least half of capillary pericytes are ASMA‐positive.^61^ We suspect that major portions of other canonically known ASMA‐negative pericyte populations across tissues might actually express this marker, and there is a possibility that ASMA is, in fact, a pan marker for pericytes that requires a more sensitive measurement technique to confirm. However, even if ASMA is expressed by all pericytes to some degree, it is not a unique marker for pericytes, because it is also expressed by vascular smooth muscle cells.

Finally, the expression of Tie2 by pericytes has been fiercely debated in the past decades, with extensive characterization of Tie2 expression in cultured pericytes,^62^ but a lack of Tie2 expression noted in pericytes in vivo.^63^ However, recent evidence has shown that a pericytes‐specific knockout of Tie2 leads to dramatically altered vascular structure, paired with a demonstration of Tie2 acting as a potent pericyte chemokine in vitro,^64^ together suggesting that Tie2 signaling may serve an important role in pericyte function. However, this finding awaits confirmation with the development of effective antibodies or other methods to directly label Tie2 in tissue and demonstrate pericyte expression in vivo. This controversy highlights the need to utilize measurement techniques that avoid such complications with variable results from tissue processing, such as fluorescent in‐situ hybridization^65^ which measures RNA expression of the target gene directly.

### 2.2 Animal models with endogenously labeled vasculature

An increasing number of transgenic murine models have been developed to visualize the microvasculature, including those that contain cell‐type specific fluorescent reporters for endothelial cells, smooth muscle cell, and pericytes (Table 2). We emphasize there is an important limitation of these reporter models that is often ignored: these animal models often only include the proximal endogenous reporter region with the fluorescent reporter, meaning that gene expression behavior from distal enhancers is often lost. An example of this is with Tie2 expression, which has been found in other cell types such as HSCs.^66^ and neutrophils, endothelial progenitor cells, macrophages,^67^ pericytes, 62 and keratinocytes.^68^ Yet the primary Tie2‐GFP mouse model is only known for GFP expression exclusively in endothelial cells,^69^ in this case serving as an advantage with a reporter line that appears to selectively label the vasculature and not track other cell types known to have endogenous expression.

**Table 2:** Animal Models to Label Microvasculature

Name	Species	Sub Pop.	Cell Type Overlap
Tie2‐GFP	Ms, Zb^200^	EC^200^	None^201^
Tie2‐Cre‐GFP	Ms	EC^202^	HSCs^203^
Tie2‐CreERT2‐EGFP	Ms	EC^204^	None^205^
VE‐Cad‐Cre‐GFP	Ms	EC^206^	Lymphatic endothelial cells^206^
VE‐Cad‐CreERT‐GFP	Ms	EC^207^	None^208^
FLT1:GFP	Ms^209^, Zb^210^	EC^210^	Migratory angioblasts^210^
FLK1:GFP, RFP	Ms^211^, Zb^212^	EC^213^	None^212^
My11‐CreERT‐YFP	Ms	PC, SMC^174^	None^175^
PDGFRB‐CreERT2‐TOM	Ms	PC,SMC^214^	Glial cells^214^
NG2‐Dsred	Ms	PC, SMC^156^	Oligodendrocyte progenitors^215^
NG2‐Cre‐ERT2M	Ms	PC, SMC^216^	Oligodendrocyte precursors^205^
Tie1‐EGFP	Ms	Embryonic EC^217^	None^217^
PDGFb‐CreERT2	Ms	ECs^218^	None^218^
β‐Actin‐GFP	Ms	EC, PC^219^	Not examined^205^
α‐SMA‐GFP, RFP^220^	Ms	Sub. PC, SMC^221^	None^221^
α‐SMA‐CreERT2	Ms	Sub. PC, SMC^222^	None^222^

EC, endothelial cell; HSC, hematopoietic stem cell; PC, pericyte; SMC, smooth muscle cell. **Species**: Ms, mouse; Zb, zebrafish.

*Note:* Labeling can vary across tissues, and in most cases not rigorously verified.

### 2.3 In vitro and ex vivo models to study the microvasculature

Over the last century, various models have been used to study the complexity of the microvasculature, including those that utilize cultures of various cell populations, tissue explants, and animal models (Table 3). Historically, researchers have had to consider significant trade‐offs when choosing between different model systems. In vivo models typically have the best chance of recapitulating human disease since pathologies are heavily influenced by the complex interplay of a multitude of cell types.^70^ However, this benefit comes at a cost: In vivo models are usually limited by throughput, exhibit high cost in time and resources, and imaging techniques restricted by limitations with sedation duration, subject restraint, and detector scan speed. Furthermore, typically any intervention short of a cell‐specific knockout of an implicated gene will not establish cellular mechanism, which can take years to generate in an in vivo model. In vitro models typically exhibit much higher throughput and have a wider range of available analysis tools to characterize the system,^71^ but at the cost of greater simplification and abstraction of tissue structure and disease conditions compared to in vivo, such as lacking various cell types or blood flow.

**Table 3:** Model Systems of Microvascular Architectures

Name/ Tissue	Type	Species	Throughput	Noninvasive Setup	Δ Vaso. Diameter	Timelapse	Adult	Angiogenesis	Network	Lumen	Flow	Mural
Chick Chorioallantoic Membrane	*In vivo*	Ch^223^	++	✗	✓	✓	✗	✓	✓	✓	✓	✓
Mesentery	*In vivo*	Ms^224^, Rt^225^, Ct^226^	+	✗	✓	✓	✓	✓	✓	✓	✓	✓
Gluteus Maximus	*In vivo*	Ms^227^	+	✗	✓	✓	✓	✓	✓	✓	✓	✓
Vessel Segments from Resistance Arteries	*Ex vivo*	Ms^228^, Hm^229^	+	✗	✓	✓	✓	✗	✓	✓	✗	✓
Skeletal Muscle	*Ex vivo*	Ms^230^, Rt^231^	+	✗	✓	✓	✓	✓	✓	✓	✓	✓
Cremaster	*In vivo*	Ms^232^, Rt^233^	+	✓	✓	✓	✓	✓	✓	✓	✓	✓
Retina	*Ex vivo*	Ms	+	✓	✗	✗	✓	✓	✓	✓	✓	✓
Embryoid Explant	*In vivo*	Zb^234^, Ms^235^, Fg^236^	++	✗	✓	✓	✗	✓	✓	✓	✓	✓
Cornea Limbal	*In vivo*	Ms^237^	+	✓	✓	✓	✓	✓	✓	✓	✓	✓
Microfluidic EC Chip	*In vitro*	Var^238^	+++	✗	✗	✓	~	✓	✓	✓	✓	✗
EC‐PC Matrix Co‐culture	*In vitro*	Var^239^, ^240^	++++	✗	✗	✓	~	✗	✓	✓	✗	✓
EC‐PC Transwell	*In vitro*	Var.^239^	++++	✗	✗	✓	~	✗	✗	✗	✓	✓
EC Culture	*In vitro*	Var.^239^	++++	✗	✗	✓	~	✓	✓	✓	✓	✗
Dermal	*In vivo*	Ms^44^	+	✓	✓	✓	✓	✓	✓	✓	✓	✓
Developing Retina	*In vivo*	Ms.^241^	+	✗	✓	✗	✗	✓	✓	✓	✓	✓
Embryoid Body	*In vivo*	Ms.^242^	+++	✗	✗	✓	✗	✓	✗	✗	✗	✓
Brain Explant	*Ex vivo*	Ms.^243^	++	✗	✓	✓	✓	✓	✓	✓	✗	✓
Retina Explant	*Ex vivo*	Ms.^244^, ^245^, Rt.^246^	++	✗	✓	✓	✓	✓	✓	✓	✗	✓
Allantois Explant	*Ex vivo*	Ms.^247^	++	✗	✓	✓	✗	✓	✓	✓	✗	✓
EC Microbeads in Fibrin	*In vitro*	Var.^248^	++++	✗	✗	✓	~	✓	✓	✓	✗	✗

EC, endothelial cell; PC, pericyte. **Species**: Ch, chicken; Ct, cat; Fg, frog; Hm, hamster; Ms, mouse; Rt, rat; Var., various; Zb, zebrafish. **Features**: yes (✓), no (✗), various (~). **Throughput**: measure of degree of throughput for each protocol. **Noninvasive setup**: if model setup requires an invasive procedure. **Δ Vaso. Diameter**: if vasoconstriction or vasodilation can practically be examined in a real‐time fashion. **Timelapse**: if system can practically be imaged continuously in a real‐time fashion. **Adult**: if tissue analyzed is from adult or embryonic. **Angiogenesis**: if angiogenesis can be observed. **Network**: if model either has existing vascular network or can form a network. **Lumen:** whether vascular structures have a lumen. **Flow**: if vascular structures exhibit flow in model system. **Mural**: if smooth muscle cells and pericytes are included.

*Note:* Table is meant to capture general trends for what is feasible in a standard version of the experiment setup, there are exceptions.

However, the trade‐offs between in vivo and in vitro models are blurring now more than ever. Advances in new imaging techniques allow for in vivo imaging that provides the opportunity for higher throughput and fully temporal measurements in various tissues. The latest in vivo gene editing techniques, such as CRISPR/Cas9,^72^ have made targeted genetic alterations easier, yet there are still challenges remaining with regards to efficiency and off‐target binding of genetic payloads.^73^ At the same time, advances in 3D bioprinting, biomaterial research, and patient‐specific primary cell culture allow for more advanced in vitro models, although there is still difficulty with cell collection in these systems for subsequent analysis.^71^ Indeed, the number of available model systems has been growing, and with the advent of new analysis techniques, the opportunities to collect data from microvascular network architecture have increased dramatically and reveal new prospects for efficient and reproducible data capture.

## 3 STATE‐OF THE‐ART IMAGING MODALITIES FOR MICROVASCULAR NETWORKS

There are a vast range of techniques available for imaging the microvasculature, with trade‐offs between resolution, signal penetration, and acquiring multiplexed functional readouts, such as blood flow and tissue oxygenation (Table 4). Beyond the classical fluorescent‐based imaging modalities that have been a mainstay for imaging microvascular structure, there now exist several new technologies that also quantify microvascular function. Advances in photoacoustic microscopy have recently enabled imaging of a wide range of tissue depots, larger fields of view, and higher resolutions, along with capturing other functional data such as blood flow velocity and tissue oxygenation.^74^ The technology behind OCT, an imaging technique based on reflected light and measuring time of flight for photons, has recently improved with resolution to the point where these imaging modalities can successfully image the microvasculature.^75^ New super resolution imaging techniques developed in the last decade, such FPALM and stochastic optical reconstruction microscopy, have allowed visualization of structures in details beyond the resolution limit of visible light, allowing for direct imaging of individual proteins and flourophores.^76^

**Table 4:** Imaging Modalities for Vascular Networks

Name	Resolution		Z Depth	Flow	Oxygen	Mechanism	Trade‐offs
	Z	XY					
Bright field	1 μm^249^	0.25 μm^249^	50 μm^38^	✗	✗	*Absorbance* 250	**+** Visualize outlines of cells^250^, low cost **+** Temporal resolution **−** Excites fluorophores outside of imaging area^250^ **−** Image is blurred by emission from out‐of‐focus regions^250^
Fluorescent Widefield	1 μm^251^	0.25 μm^251^	50 μm^38^	✗	✗	*Fluorescence* 250	+ Temporal resolution: milliseconds^252^, low cost **−** High resolution requires immersion objectives^251^ **−** Image is blurred by emission from out‐of‐focus regions^250^
Point Scanning Confocal	0.50 μm^251^	0.18 μm^251^	100 μm^253^	✗	✗	*Fluorescence* 252	+ Pin‐hole reduce out‐of‐focus light^250^ + Temporal resolution: milliseconds^252^ **−** Phototoxicity, photobleaching^254^
Spinning Disk Confocal	0.75 μm^254^	0.25 nm^254^	100 μm^38^	✗	✗	*Fluorescence* 252	+ Higher FPS compared to point scan^250^ + Less phototoxic to cell, less photobleaching^250^ **−** Poorer filtering of out‐of‐focus light compared to point scan^250^ **−** Lower resolution, smaller FOV^254^
Two Photon	0.4 μm^251^	0.20 μm^251^	1 mm^255^	✗	✗	*Fluorescent* 256	+ Useful for thick specimens (>200um)^250^ + Bleaching limited to imaging plane^251^, low light scatter^256^ + NIR light less phototoxic than VIS **−** Enhanced heating from NIR light **−** Broader excitation, pronounced photobleaching^257^
Photo‐acoustic	1 μm^258^	10 μm^258^	5 mm^259^	✓	✓	*Flow, oxygenation* 260	+ High contrast and spatial resolution, high framerate^260^ + Imaging thick tissues (>1 cm)^260^ **−** Increased resolution at expense of ultrasonic penetration^260^ **−** Comparably low resolution^260^
Laser Doppler		1 μm^261^	1 mm^261^	✓	✗	*Flow* 262	+ Live imaging of flow^262^ **−** Long mapping time^262^
Laser Speckle		10 μm^263^	1 mm^264^	✓	✗	*Fluorescence* 265	+ Resolution adequate for low‐flow microvasculature^265^ + Noninvasive, live imaging^265^, real‐time changes in flow^265^ **−** Requires knowledge of blood velocity distribution^262^ **−** Motion artifacts^265^
Second Harmonic	2.5 μm^266^	0.70 μm^266^	300 μm^267^	✗	✗	*Auto‐fluorescence* 268	+ Three‐dimensional resolution^269^, NIR wavelength + Label free^267^, Long imaging times^269^ **−** Low image quality in deep tissue^270^
Optical Coherence Tomography	2 μm^271^	1 μm^271^, ^272^	2 mm^253^	✓	✗	*Reflectance* 273	+ Temporal resolution: seconds^252^, label free^274^ + Technology contained in endoscopes, handheld probes^271^ **−** Angiography visualizes only flow, not leakage^274^
RESOLFT, STED	50 nm^250^	30 nm^275^	300 μm^276^	✗	✗	*Fluorescence* 252	+ Video‐rate, live‐cell imaging^252^ + Temporal resolution: seconds^252^ **−** Requires highly‐stable fluorophores^252^
FPALM	50 nm^250^	20 nm^275^		✗	✗	*Fluorescence* 252	+ Fast framerate for high resolution^252^, live‐cell imaging^252^ + Image single molecules/single particle tracking^252^ **−** Requires photo‐switchable fluorophores^252^
Electron Microscopy	8 nm^277^	1 nm^252^	<1 μm^278^	✗	✗	*Fluorescence* 252	+ High resolution^252^ **−** Limited labeling options^19^, no temporal resolution^252^, ^279^ **−** Restrictive sample prep.^252^, ^279^
Light‐Sheet	0.75 μm^280^	0.25 μm^280^	10 mm^264^	✗	✗	*Fluorescence* 280	+ Excellent optical sectioning 3D imaging^281^ + Low bleaching and phototocicity^281^ **−** Restrictive sample prep^281^

## 4 MICROVASCULAR NETWORK ANALYSIS AND QUANTIFICATION

The microvascular network forms a sprawling architecture of interconnected vessels that vascularize nearly all tissues in the body. Such complex spatial networks undergo remodeling in adult tissue as well as embryonic; in quiescence as well as pathologic. Understanding changes in vessel morphology cannot be captured by a single metric to quantify its structure: A range of metrics must be used to summarize various unique characteristics of the network. While previous work has developed a multitude of metrics for quantifying microvascular architecture, we believe that further work must be done in both developing new metrics and demonstrating that a given set of available metrics provide unique and useful information to answer biological questions. To this end, a series of suggestions are proposed to increase scientific rigor and reproducibility of quantifying the complexities of the microvasculature.

### 4.1 Metrics for quantifying microvascular networks

**Figure 3:**
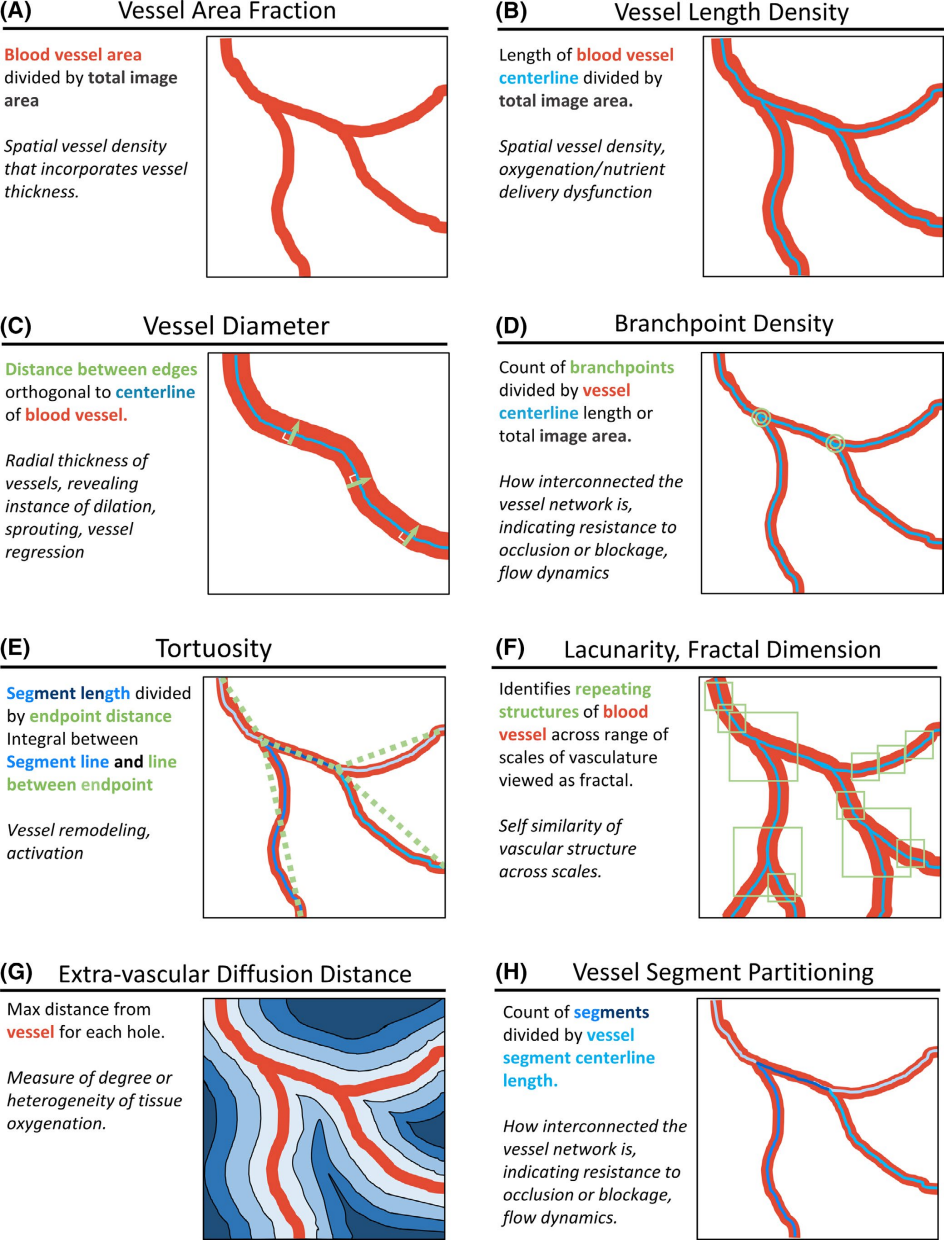
Basic metrics quantifying the complexities of the microvascular architecture. Visual explanation of metrics that have been used to quantify various aspects of microvessel network architecture, including (A) VAF, (B) vessel length density, (C) vessel diameter, (D) branchpoints density, (E) tortuosity, (F) lacranuity and fractal dimension, (G) extra‐vascular diffusion distance, and (H) vessel segment partitioning

Previous research has developed various metrics for microvascular network analysis (Figure 3A‐G), including the fraction of image area composed of blood vessels (VAF),^77^ blood vessel length normalized by image field of view (vessel length density),^78^ average vessel diameter of all vessels or divided by vessel type,^79^ density of branchpoints,^80^ tortuosity,^81^ lacranuity,^78^ fractal dimension,^81^ and max extra‐vascular diffusion distance to examine tissue oxygen perfusion.^82^ Other metrics have been developed outside of this set but not standardized and adopted by consensus. Studies often normalize metrics in different ways, such as measuring branchpoints per image, normalizing to field of view, or normalizing to vessel length. We posit that metrics should be designed to encourage valid comparisons across research studies and should be normalized to facilitate this process. Thus, using a simple metric of vessel length^83^ is not as useful as vessel length density, a metric that can be directly compared over a range of spatial resolutions and imaging modalities.

### 4.2 Architectural features of microvasculature: bridging form and function

**Figure 4:**
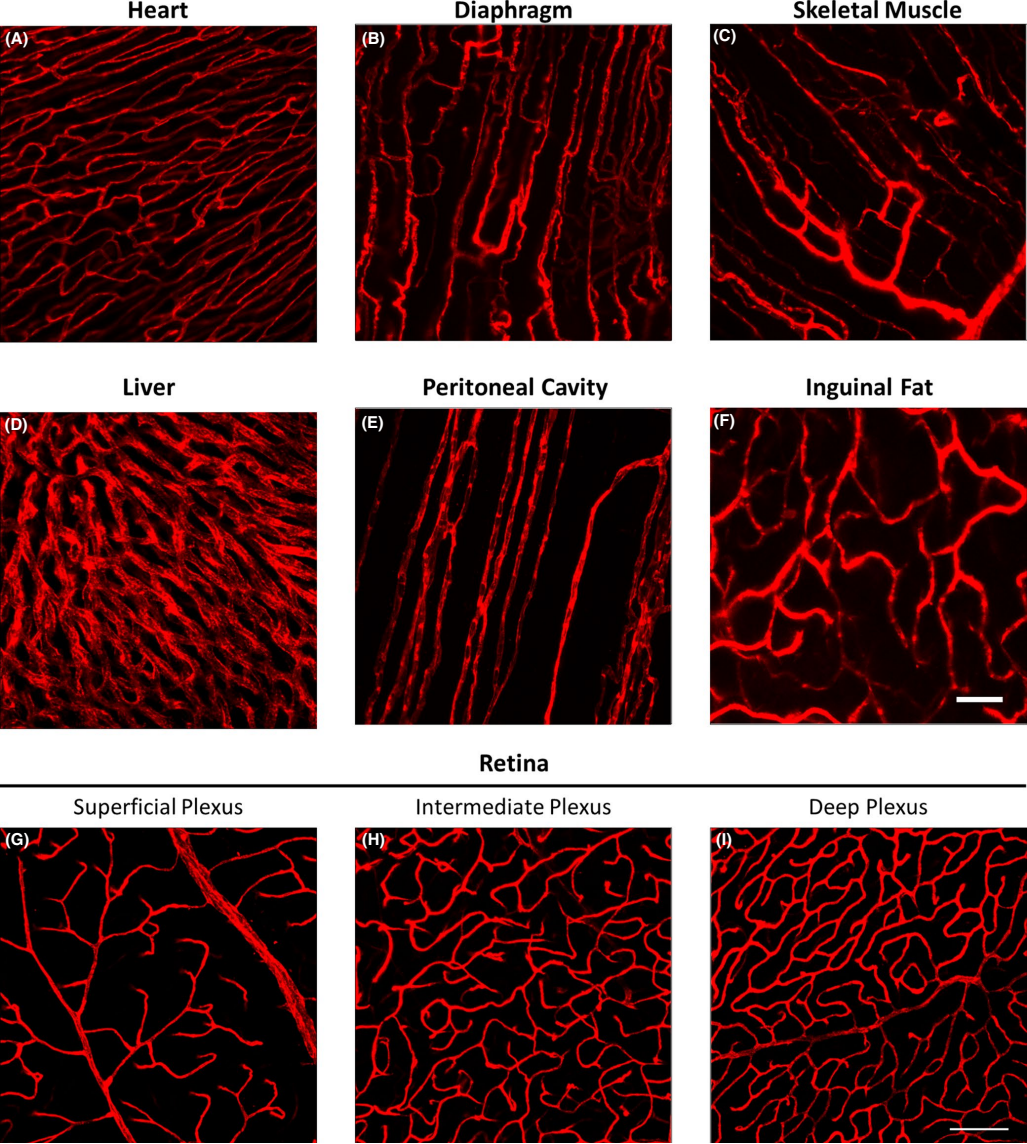
Heterogeneity of blood vessel network structure across and within tissue. IB4 lectin Perfused microvessels of (A) heart, (B) diaphragm, (C) skeletal muscle, (D) liver, (E) peritoneal cavity, (F) inguinal fat, and of the three distinct vascular layers of the retina (G‐I; scale bar 50 μm)

Capillary architecture possesses markedly different structures to meet the unique metabolic demands of peripheral tissues,^84^ including the radial spoke‐wheel structure of the retina, parallel beds of skeletal muscle, or dense networks of the liver (Figure 4A‐F). Even within a single tissue such as the retina, there is impressive heterogeneity in microvascular architecture between tissue locations (Figure 4G‐I). This tissue environment heterogeneity is further reflected by unique endothelial transcriptomes found in each organ^85^ and distinct endothelial marker profiles at different parts of the vascular tree.^86^

**Figure 5:**
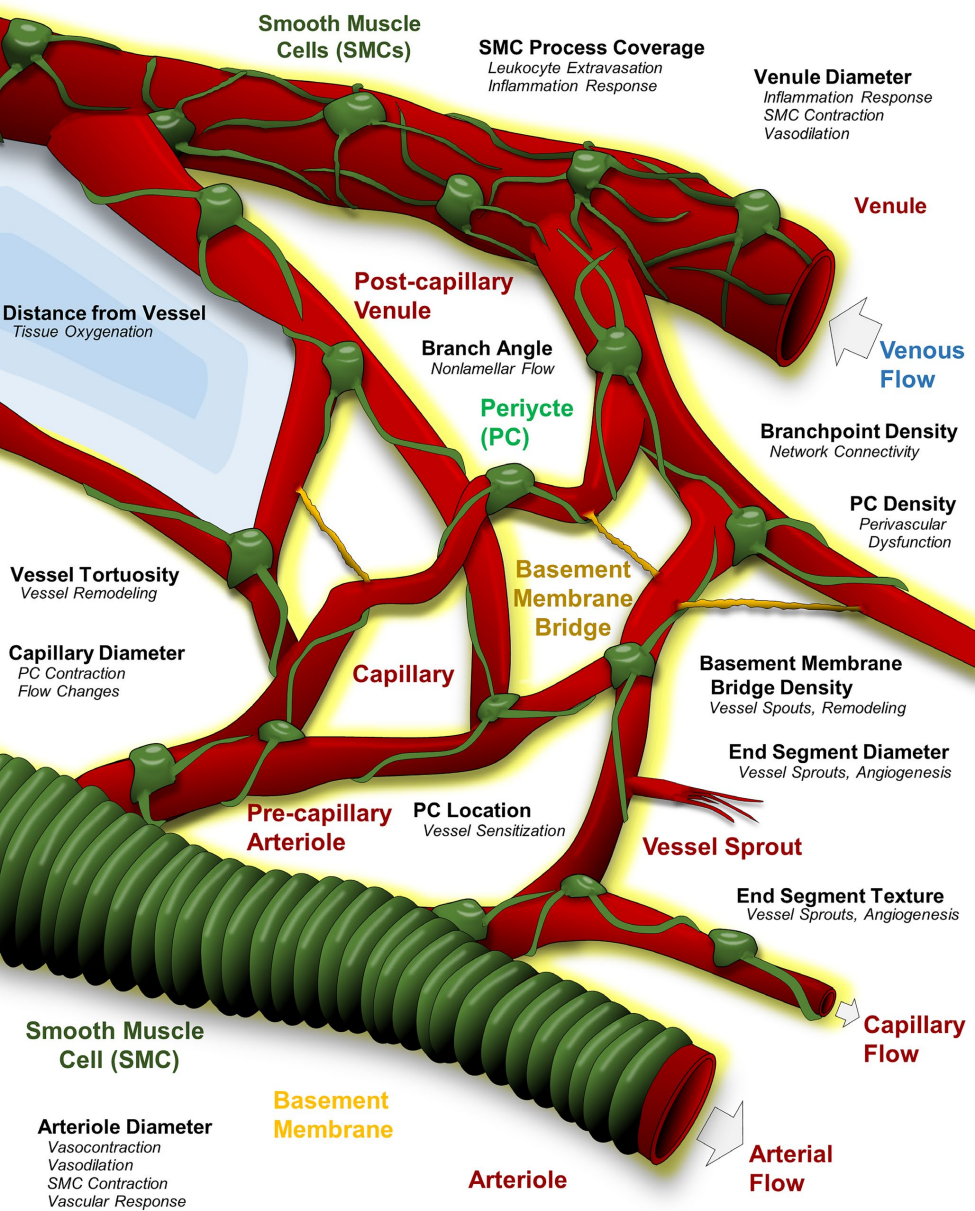
Bridging form and function: correspondence between microvasculature architecture metrics and biological behaviors. Schematized microvasculature network with various cellular and acellular components (multicolored font) mapped to quantitative image analysis metrics that indicate different aspects of microvascular function (black font)

With much of biology, function and form are closely interwined^87^: the microvasculature is no exception. A wide range of biological behaviors, including blood vessel growth, regression, dilation, constriction, stability, and permeability, can be mapped to quantitative metrics of microvascular structure and give insight into physiologic and pathological function of the microcirculation (Figure 5). Beyond the adaptations of microvessel networks to support unique tissue metabolic environments, morphological changes in vessel structure are hallmarks of key vascular remodeling events. Spatial distribution of capillary networks determines spatial heterogeneity of oxygenation and nutrient delivery.^88^, ^89^ Enriched build‐up of extra‐cellular matrix can indicate a fibrotic response^90^ to inflammatory conditions. Increased blood vessel tortuosity can indicate signs of endothelial cell activation and pathological microvascular remodeling and/or ischemia‐induced arterialization in collateral microvessels.^91^

Moreover, certain quantification metrics may carry unique significance depending on the location analyzed within the vascular tree. For instance, changes in vessel diameter in arterioles suggest changes in vascular smooth muscle cell vasoconstriction to modulate vascular resistance,^92^ capillary diameter changes are indicative of changes to pericyte contraction and distribution of flow and oxygenation,^93^ and venule dilation suggests changes to blood capacitance (storage)^94^ or remodeling in response to inflammation to facilitate leukocyte extravasation from circulation.^95^

### 4.3 The need for pairing perivascular and microvascular analysis

Analyzing changes to the perivascular space can yield just as important insights as the vasculature itself given the close cross‐talk between endothelial cells and smooth muscle cell and pericytes. For example, changes in pericyte density are known to play a key role in the pathogenesis of diabetes,^27^ and changes in pericyte locations relative to branchpoints^96^ have been associated with changes to stability and sensitization of the microvascular network. We emphasize the need to analyze perivascular behavior as well as microvascular remodeling to truly understand the structure and function of microvascular networks: A common limitation of many studies is to study one or the other in isolation. Most existing software for quantifying microvascular network architecture accomplishes this by evaluating the endothelial network structure. There is one software package that can analyze perivascular cell recruitment to the vasculature through overlap of the two structures.^97^ While this metric may be confounded by changes in perivascular or vascular density across study groups, and perivascular cells may associate with the vasculature with minimal channel overlap, this software allows researchers to begin to probe perivascular interactions with the microvasculature. A survey of the published literature reveals that the most common approach for analyzing perivascular cell coverage and/or morphology (eg, of pericytes,^98^ smooth muscle cell,^99^ or other cell types like macrophages^100^ that are known to play key roles in angiogenesis and vessel remodeling^101^) is through manual or basic automated comparisons of thresholded area or nuecleii.^102^ For example, basic cell counts can be obtained manually through ImageJ's cell^103^ counting feature, the multipoint tool,^104^ or using its particle analysis set of tools for automated analysis.^104^ More extensive positional or morphological investigations of perivascular structures require custom image analysis solutions that have yet to be developed. Automated and quantitative analysis of microvascular networks paired with detailed analysis of smooth muscle and pericyte cell populations could become a standard pipeline that would enable better understanding of microvascular remodeling mechanisms and the development of new therapeutics for microvascular diseases.

### 4.4 Software packages for quantifying microvascular networks

Alterations in microvessel network architecture have been used ubiquitously in studying vascular diseases, and there are a multitude of software packages available for quantifying changes in architecture (Table 5). Three, notably, have been used in a significant number of publications, namely AngioQuant, AngioTool, and RAVE.

**Table 5:** Vessel Network Analysis Tools

Name	Lang.	Application	Metrics	Validation	Cts	Yr	Cts/yr
AngioQuant^83^	MATLAB	Brightfield, Cell Culture	Segment Count, VL, VA, VAF, Segment Area, BP, BP/Segment Count	QT BD	104	2005	8
RAVE^81^	MATLAB	Fluorescent, Tissue	VAF, VLD, VR, FD	QT manually analyzed BD for VAF, VLD, and VR. Basic *In silico* for FD	24	2011	3.4
Vessel Analysis ImageJ Macro	ImageJ Macro	Brightfield, Tissue	VAF, VLD	None	‐	‐	‐
AngioTool^78^	Java, ImageJ	Fluorescent, Tissue	VAF, SC, VL, VLD, EP, lacunarity, BP/area	QL manually analyzed BD	111	2011	15.9
CSAQ^282^	*Web Based*	Brightfield, Tissue	VL, VAF, BP; Contrast, Entropy	Manual (BP), QT comparison to AngioQuant	24	2008	2.4
Breast Tumor Microvasculature Reconstruction^82^	MATLAB, JAVA	Micro Ct, Tumor	VLD, VD, SL, MEVDD, FD, Tortuosity, Flow	Metrics from BD within literature values	17	2013	3.4
VESGEN 2D^283^	Java, ImageJ	Brightfield/ Flourescence, Tissue	VD, VT, FD, VAF, VLD, BP	QT BD	29	2009	3.2

BD, biological data; BP, branchpoints; EP, endpoints; FD, fractal dimension; MEVDD, max extra‐vascular diffusion distance; NS, not specified; QL, qualitative; QT, quantitative; SC, segment count; SL, segment length; VLD, vessel length density; VD, vessel diameter; VR, vessel radius.

AngioQuant has been developed to analyze endothelial networks in vitro, with a focus on quantifying various metrics of tubule formation using bright field images.^83^ Recently, it has been adapted for use in evaluating higher resolution datasets of microvascular networks in vivo^105^ and in histological samples.^106^ Its validation is focused on quantification of in vitro experiments showing trends of changes with various metrics, but no statistical comparisons between study groups. The datasets were not validated against manually analyzed images and there is no analysis included comparing accuracy and overall performance between AngioQuant and other available software packages.

AngioTool is presented as a quick, hands‐off, and reproducible image analysis tool, deployed as an ImageJ plugin, for quantification of microvascular networks in microscopic images.^78^ The validation of AngioTool included analyzing biological data from murine hindbrain and retina using various metrics, including visualized vessel segmentation, vessel centerline, and branchpoints presented for qualitative inspection. For quantitative biological validation, endothelial cell explants were cultured and analyzed with two drug treatments that were known to alter vascular structure as a positive control.^78^ Additionally, output metrics were validated with a subset of manually counted images in an unblinded fashion with two investigators.

RAVE is an image analysis tool that can be used on a wide array of images^81^ to accelerate the unbiased, quantitative analysis of microvasculature architecture. Validation for this tool included comparing automated outputs generated by RAVE to manual analysis of various morphometric parameters for in vivo microvessel networks in murine spinotrapezius muscle tissue and in a xenograph tumor model. A dataset of images was compared to manually processed images with a Bland‐Altman analysis.

### 4.5 Improving vessel quantification, analysis, and interpretation

We estimate that these software packages are largely underutilized, based on the high number of published manuscripts that refer to the quantification of microvessel architecture. Indeed, a search on PubMed on relevant terminology (terms used included: microvasculature density, capillary dropout, pericyte dropout; see all terms in Appendix S1) reveals over 120 000 publications to date. While this query includes publications that merely mention the terms searched for, the nearly three order‐of‐magnitude difference between citations of these software programs compared to this large collection of publications suggests that there is an unmet need for vessel architecture analysis beyond the available options, with researchers often resorting to manual ad hoc analysis of microvessel networks, leading to decreased repeatability, comparability, and scientific rigor. We propose the following design criteria for an effective software package:

- **Ground truth validation:** A rigorous and complete validation of software requires a comprehensive analysis of multiple types of datasets. This includes an extensive comparison of automated results to manually processed images, not just with output metrics, but also with the pixel‐by‐pixel raw segmentation, skeleton centerline, and branchpoints locations quantified with a combination of false positive rates, false negative rates, Bland‐Altman analysis, and SSR. Ideally, this manual comparison would include multiple study groups with known vascular differences in architecture between them.
- **Biological validation:** Validating the automated pipeline with several biological datasets with known differences (biological positive controls), ideally from different tissue and/or imaging resolutions, will demonstrate the efficacy of the program in practice. This analysis demonstrates that the program can detect true positives in actual dataset, where a real change is detected between study groups.
- **In silico validation:** Program development needs to be paired with a validated parameterized computational model that can generate artificial in silico vascular networks^107^ to verify that changing basic parameters of the model yields expected changes in metric output with the image analysis pipeline.
- **Quantitative comparisons between previously developed programs:** The field benefits far less from releasing another “one‐off” vessel image analysis program independent from previous work and instead should test and demonstrate its efficacy compared to existing software. Outputs from each program and manual analysis should be compared, including output metrics, raw segmentation, skeletonization, and branchpoints assignment in the form of false positive and negative error rates, Bland‐Altman analysis, and SSR. Execution time should also be critically evaluated and reported, given the importance of balancing throughput with accuracy.
- **Standardized metric sets:** Each software program has a unique collection of metrics that are often calculated using different methods. A consensus of metrics needs to be established in the interest of rigor and reproducible science. An example of this would be measuring branchpoints: Some packages display raw counts, while others normalize to image area or vessel length.
- **Effectiveness of metric collections:** Methods should be developed to not only test and validate each method but also demonstrate their usefulness as a collection in determining changes in the vascular architecture. Analysis needs to be done to show that these new metrics provide unique non‐correlative information compared to existing metrics, utilizing techniques developed from the field of feature selection.^108^
- **Effect of image quality on output metrics:** An examination needs to be performed with respect to how the program performs when image quality varies between study groups from batch effects, or simply has a high degree of variance. Image quality of the datasets provided with these software packages often appears ideal. High variance with image quality may skew segmentation results and output metrics, so output metrics across a range of image qualities should be examined.
- **Effect of parameter adjustment:** Some software packages allow for adjustment of key image processing parameters to enhance results, but the effect (and bias) of allowing the user to freely alter image processing outcomes needs to be rigorously examined and reported.
- **Blinded image analysis:** Software packages should include built‐in support for image filename anonymization to blind the user from an image's study group assignment to minimize bias as images are analyzed.
- **Semi‐automated curation:** Image quality and marker expression can change between study groups, which could bias automated segmentation and results. Image analysis programs should, therefore, build on previous efforts^97^ to include the option to efficiently curate segmentation within a study group in a blinded fashion in regions where automated analysis fails. While some of the programs allows for a degree of curation with adjusting image processing parameters, there is no examination with how this can bias results if the researcher changes these parameters between study groups or images.
- **Built‐in detection of insufficient sampling:** It is important for the image datasets to sample enough of the microvasculature for valid metric quantification. For simple metrics, such as vessel length density or VAF, a simple examination of variance and power analysis can determine whether more images of a biological sample are required. However, for more advanced quantification metrics, such as lacunarity and fractal dimension, or graph theory‐based metrics, the metrics become nonsensical if the field of view is too small and not enough vascular network sampled. These metrics need to be studied and predictive algorithms developed to warn the user if the dataset is at risk for yielding invalid results for advance structural metrics.
- **Source code and dataset availability:** While most published software packages will provide source code and data upon request, we believe that it should be standard to make both freely available for download on long‐term hosting platforms such as Github, Bitbucket, or an institutional repository. This removes any barrier to iterate on previous work and facilitates comparison of software packages. Freely available image datasets will also standardize the validation process and enhance progress within the field in the same way standard datasets have with analyzing retina microvessels in fundus imaging.^109^, ^110^
- **Novel metrics:** More metrics need to be developed to describe all observed complexities found in the network structure, with the long‐term view that a sufficient number of metrics for characterizing the microvasculature would allow for the successful creation of in silico artificial networks that are indiscernible from experimentally derived microvascular network structures. Any degree short of this reproduction would mean that information is being lost by the current metric set. Techniques for developing new metrics can be guided from the field of feature engineering.^111^

Without tackling these issues, new software programs are merely presented to research scientists “as is” without allowing them to make informed decisions on how to produce high‐quality unbiased results. We argue that until these issues are dealt with, the use of these packages has the risk of leading to a significant error rate in microvascular research: where the software reveals a positive error with a quantifiable change between groups where none existed, or even worse, where research is not pursued based on a negative error where no change is observed between study groups where one exists.

### 4.6 Applications of machine learning, graph theory, and modeling in quantifying microvascular architecture

Metric effectiveness not only needs to be evaluated on an individual basis, but the effectiveness of a given collection of metrics in combination needs to be evaluated. This can start with examining covariance matrices of metrics from in silico and biological datasets to evaluate how much unique information they bring relative to one another. Effectiveness of metric sets could be evaluated using principal components analysis, partial least squares, or more advanced methods of feature selection techniques from the field of feature engineering,^108^ especially when applied to in silico artificial networks where basic parameters (vessel length density, vessel diameter, branchpoints density, tortuosity, connectivity) can be changed in a controlled fashion. Further research must be conducted on what makes an effective metric in an unbiased fashion, and consensus much be reached on normalization of metrics so they can be used in a standardized way, such as the disagreement on how to normalize branchpoints counts (by image area, vessel length, or binned vessel diameter). This will depend on the development and pairing of fully parametrized in silico models^112^ (Table 6), structural models of models of vascular flow,^113^, ^114^ and models of predictive tissue oxygenation^115^ with biological experiments^116^ to fully connect metrics of microvascular structure to biological behaviors.

**Table 6:** Computation Models of Microvascular Architecture

Description	Language	Method	Type	Validation
Particle‐based EC Network^284^	C++	ABM, CPM	Vasculogenesis	QT to classic CPM
Retina ABM^285^	NetLogo	ABM	Retinal developing vasculature	QT comparison retinal BD
3D CPM of Tumor Growth^286^	CompuCell3D	ABM, CPM	Tumor angiogenesis	QL to Macklin et al.^287^
ABM for Disruption of Vasculogenesis^288^	CompuCell3D	ABM	Vasculogenesis	QT to BD with AngioTool^78^
3D Sprouting Angiogensis^289^	NS	ABM	Sprouting angiogenesis	QT metrics within BD range
Hypoxic Vessel Sprouting^290^	NS	ABM Hybrid	Any 2D or 3D vessel formation in a tissue	QT comparison with BD
Tumor Angiogenesis and Patterning^291^	NS	ABM Hybrid	Sprouting angiogenesis	QT metrics within BD range
Angiogenesis with Discrete Random Walks^292^	NS	ABM Hybrid	Tumor angiogenesis	QL assessment of simulation results
ABM of Tumor Angiogenesis and Regression^293^	NS	ABM Hybrid	Tumor angiogenesis and regression	QL to BD
3D Phase Field ABM of Vascular Networks^294^	NS	ABM Hybrid	3D Angiogenesis	QL to basic theoretical behavior
Adaptive Network with Flow^295^	C	Stochastic Hybrid model	Flow, oxygen transport, and adaptation of existing network	Thorough quantitative comparison to experimental results across all categories
Multiphase Tumor Angiogenesis Growth^296^	CAST3M	Continuum Discrete	Tumor growth	QT comparison with BD
Vessel Generator for Cell‐colocalization (CIRCOAST)^135^	MATLAB	Structural Descriptive Model	Static adult microvascular network for basic model validation	QT metrics within BD range
Tumor Angiogenesis with Blood and Interstitial Flow^297^	NS	Hybrid	Tumor growth	QT metrics within BD range

ABM, Agent‐based model; BD, biological data; CPM, cellular pots model; QL, qualitative; QT, quantitative; NS, not specified.

**Figure 6:**
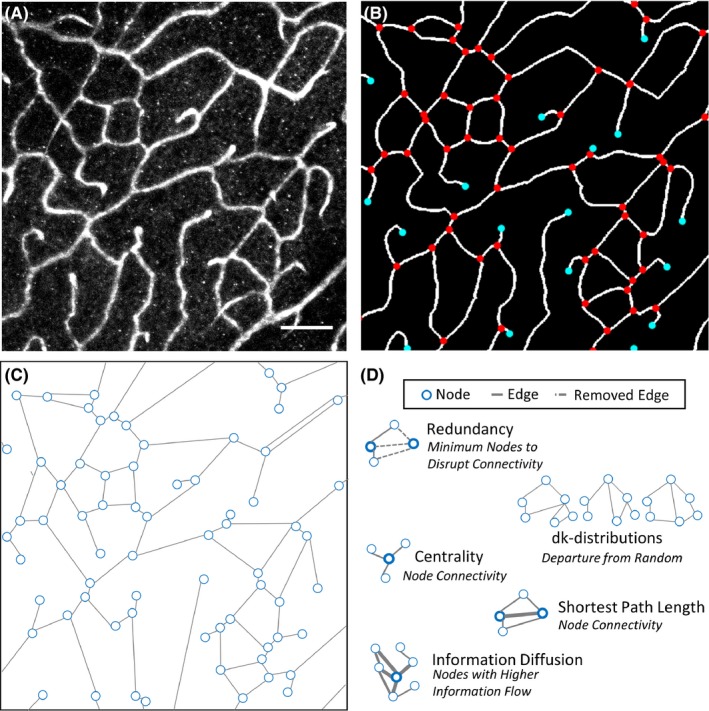
Novel image analysis metrics by analyzing the microvasculature with graph theory. A, Confocal microscopy image of the murine retinal deep vasculature, with CD105 marking ECs (white; scale bar = 50 μm). B, Image analyzed with basic segmentation, skeletonization, and branchpoints classification, with vessel centerline/skeleton (white), branchpoints (red), and EP (turquoise). C, Conversion of vasculature into a graph, with branchpoints indicated as “nodes” (blue) and vessel segments as “edges” (gray). D, Visual summary of types of metrics to quantify graphs, with removed edges representing change to network after a blood vessel has regressed or experiences obstructed flow

Blood vessel networks can be abstracted as a series of branchpoints, or nodes, with varying connectivity with each other. Previous work has explored the basic concept of abstracting the microvasculature as a graph,^117^ and we believe there are a wealth of relevant metrics that could be applied from quantifying graph networks (Figure 6). Examples include metrics measuring centrality of each node,^118^ scoring the relative importance of edges in connecting nodes, connective redundancy,^119^ and information diffusion.^120^ Machine learning methods such as convolution neural networks and other techniques^121^ have also been applied to graph networks.^122^ Previous applications of network tomography outside of biomedical research,^123^ such as internet tomography,^124^ may be a pertinent source of applicable methods for characterizing complex microvascular networks.

### 4.7 Relevant techniques from related network architectures

Analysis of full feature microvascular architectures can also benefit from adapting techniques used to analyze similar network structures. A prime example is fundus imaging of human retinal vascular networks: Although such images fail to resolve capillary portions of the microvascular network, this application has been extensively explored due to its established clinical significance in evaluating eye disease and a collection of systemic diseases,^125^ with a plethora of methods developed to segment, quantify, and validate vessel architecture.^126^ Indeed, a machine learning‐based classification pipeline for fundus images has recently been approved by the FDA for diagnosis of diabetic retinopathy.^127^ Methods used in the processing and quantification of neuronal network image datasets^128^ may yield useful techniques for analyzing microvascular networks.^129^ In vivo clinical imaging techniques, such as micro‐CT^130^ of lung vascular networks, may also yield insight into extending 2D imaging and quantification into the third dimension.^131^

Although this review focuses on 2D image quantification techniques, many of the imaging modalities mentioned acquire data in 3D directly, or through a series of 2D slices. Over the long term, the field would most benefit from acquiring and analyzing 3D datasets to eliminate any confounding phenomena that arises from analyzing a 2D‐projected representation of complex 3D structures. Such 2D abstractions can lead to altered metrics, such as false branch points where vessels appear to overlap in 2D but exist at distinct elevations in 3D, introducing error to other metrics such as segment length. Furthermore, the 3D orientation of vessel segments relative to one another is especially important when characterizing local tissue oxygenation.^89^ An in‐depth examination of how 2D structural metrics can characterize a projected 3D structure like a microvascular network would also be necessary to understand the trade‐offs and reveal what information is missed with this simplification.

### 4.8 Statistics to analyze microvascular interactions: beyond generic

The metrics covered in this review require basic 2‐sample or multi‐sample statistical tests to determine whether there is a difference in structure and morphology of vascular networks between study groups. Yet there are also new statistics being developed based on modeling null distributions (output if the null hypothesis is true and there is no difference between groups) that could be extended to quantifying the microvascular architecture. A prime example of this is a technique to measure cellular recruitment with a given cell type and the vascular network,^132^ that maintains validity in conditions where generic statistics fail. While cell recruitment has been analyzed,^97^ previous metrics of cell‐to‐cell colocalization events fails to properly measure changes in cell recruitment if there are changes to vascular density or cell density across study groups. This confounding phenomenon will lead to false positives when testing between study groups^132^: instances where the test indicates there is a significant change in cell recruitment, when in reality there is none. Null modeling of random cell placement is used to avoid the deficiencies of generic statistics, and we believe this modeling approach could be applied to evaluating perivascular cell recruitment to blood vessel architecture using an in silico model and provide researchers with a more robust statistical hypothesis test for analyzing microvessel architecture.

## PERSPECTIVES

The microvasculature is implicated in pathogenesis and maintenance of the deadliest maladies of the modern world. Understanding microvasculature's function, adaptation, and contribution to disease is enabled by the application of metrics that quantify changes to microvascular network architecture in in vivo, in vitro, and in silico model systems. We highlight opportunities to further the field by improving scientific rigor and reproducibility through the development and validation of software that reliably, comprehensively, and in an automated manner, characterizes the complexities of microvascular architecture using pre‐existing and novel metrics.
