# Supplementary material for: Methods to label, image, and analyze the complex structural architectures of microvascular networks
Source: Microcirculation. 2019 Jan 17;26(5):e12520. doi: 10.1111/micc.12520 (PMC6561846; doi:10.1111/micc.12520)
Supplement: Supplementary file 1 [file MICC-26-na-s001.docx]

**Methods to Label, Image, and Analyze the Complex Structural Architectures of Microvascular Networks**

Bruce A. Corliss^1,*^, Corbin Mathews^1^, Richard Doty^1^, Gustavo Rhode^1^, Shayn M. Peirce^1^

^1^Department of Biomedical Engineering

415 Lane Road

University of Virginia

Charlottesville, VA 22908

***Corresponding Author**:

Bruce A. Corliss

Ph.D Candidate

Department of Biomedical Engineering

415 Lane Road

University of Virginia

Charlottesville, VA 22908

**Supplement Contains** –

Supplemental Note 1

Supplemental Methods

## Supplemental Note 1

Pubmed Query for Relevant Literature (Performed 8/3/2018): 193707 Publications

"microvascular density" OR "vessel density" OR "endothelial cell density" or "capillary density" OR "blood vessel density" OR "vessel fraction" OR "vessel drop-out" OR “capillary drop-out” OR “vessel regression” OR "vessel diameter" OR "arteriole diameter" OR "venule diameter” OR “vessel remodeling” OR “Vascular remodeling” or “arteriole remodeling” OR “angiogenesis” OR “arteriogenesis” OR “vasculogenesis” OR vasoconstriction OR vasodilation

## Supplementary Methods

### Mice

All procedures were approved by the Institutional Animal Care and Use Committee at the University of Virginia, and completed in accordance with our approved protocol under these guidelines and regulations. We used C57Bl6/J mice from The Jackson Laboratory (JAX stock #000664, Bar Harbor, ME). For mice with perfused lectin, animals were anesthetized with an intraperitoneal injection of ketamine/xylazine/atropine (60/4/0.2 mg/kg body weight) (Zoetis; Kalamazoo, MI/West-Ward; Eatontown, NJ/Lloyd Laboratories; Shenandoah, IA). A drop of sterile 0.5% Proparacaine hydrochloride ophthalmic solution was added as a topical anesthetic to numb the eye before injection. To allow visualization of vascular endothelium, anesthetized mice were administered a retro-orbital injection of labeled isolectin (IB4-Alexa647; Life Technologies, Carlsbad, CA) 30 minutes before sacrifice.

### Tissue Preparation

Mice were sacrificed via CO2 asphyxiation with cervical dislocation for secondary sacrifice, eyes enucleated, and incubated in 4% PFA for 10 minutes. Lectin perfused retinas were isolated, flat-mounted, and sealed on a microscope slide. Otherwise, retinas were immunostained as described previously^1^. Primary antibodies used included NG2 (1:200, AB5320, Millpore, Darmstadt, Germany), anti-collagen-IV (1:100, 13400, Bio-rad, Oxford, United Kingdom), IB4 lectin (1:150, I32450, Life Technologies, Carlsbad, CA), and DAPI (1:500, D1306, ThermoFisher Scientific, Waltham, MA). Secondary antibodies used were donkey anti-rabbit 546 (1:600, A10040, Invitrogen, Carlsbad, CA), and donkey anti-ret 647(1:600, Ab150155, Abcam, Cambridge, United Kingdom).

### Imaging

Images of perfused mice retina were acquired on a Nikon point scanning confocal (Nikon Instruments Incorporated, Melville, NY; Model TE200-E2; 20X air objective). Images of immunostained mice retina were acquired on a Zeiss CLSM confocal (Zeiss CLSM 880, 63x oil objective).

### References

1. Tual-Chalot S, Allinson KR, Fruttiger M, Arthur HM. Whole Mount Immunofluorescent Staining of the Neonatal Mouse Retina to Investigate Angiogenesis In vivo. 2013 [accessed 2018 Aug 30];(77). https://www.ncbi.nlm.nih.gov/pmc/articles/PMC3732076/. doi:10.3791/50546
